# Digital adaptive optics based on digital lateral shearing of the computed pupil field for point scanning retinal swept source OCT

**DOI:** 10.1364/BOE.416569

**Published:** 2021-02-23

**Authors:** Abhishek Kumar, Stefan Georgiev, Matthias Salas, Rainer A. Leitgeb

**Affiliations:** 1 Center for Medical Physics and Biomedical Engineering, Medical University of Vienna, Austria; 2 Wavesense Engineering GmbH, Vienna, Austria; 3 Vienna Institute for Research in Ocular Surgery, Austria; 4 Christian Doppler Laboratory for Innovative Optical Imaging and its Translation to Medicine, Medical University of Vienna, Austria; 5 These authors contributed equally to this work

## Abstract

A novel non-iterative digital adaptive optics technique is presented in which the wavefront error is calculated using the phase difference between the pupil field and its digital copies translated by a pixel along the horizontal and vertical direction in the pupil plane. This method provides slope data per pixel, thus can generate > 50k local slope data samples for a circular pupil of diameter 256 pixels with high accuracy and dynamic range. It offers more than 12x faster computational speed in comparison to the sub-aperture based digital adaptive optics method. Furthermore, it is independent of any system parameters, the light distribution in the pupil plane, or the intensity of the image. The technique is useful in applications such as interferometric or digital holography based microscopy, metrology, and as digital wavefront sensor in adaptive optics, where focusing of light in the sample is involved that creates a guide star or where the sample itself exhibits guide star-like structures. This technique is implemented in a point scanning swept-source OCT at 1060 nm, and a large wavefront error with a peak to valley of 20 radians and root mean square error of 0.71 waves is detected and corrected in case of a micro-beads phantom sample. Also, human photoreceptor images are recovered from aberrated retinal OCT volumes acquired at eccentricities of 2 and 2.5 degrees from the fovea *in vivo*.

## 1. Introduction

OCT is a well-established technique used for imaging of anterior and posterior segment of the eye for clinical diagnosis of ocular disease [1,2]. High-resolution cross-sectional images with axial resolution, which depends on the bandwidth of the light source, of typically ∼5 µm in tissue is possible with commercially available OCT devices. However, the lateral resolution, which depends on the numerical aperture (NA) or the beam diameter at the eye pupil, is conventionally kept low at typical ∼20 µm in order to have a uniform lateral resolution over a large depth of field of > 250 µm that can typically cover the imaging depth of interest in the tissue. Hardware based adaptive optics (HAO) has been combined with OCT in order to achieve cellular level lateral resolution enabling close to diffraction limited imaging of photoreceptors, micro-vasculature and recently ganglion cells [3–6]. The availability of OCT images of the retina with cellular level resolution in all three dimensions can potentially allow researchers and clinicians to investigate the genesis and development of ocular disease as well as treatment success at the cellular level.

OCT being an interferometry-based imaging technique provides phase information in addition to the amplitude of the signal. This has been exploited by researchers who demonstrated that computational or digital post-processing techniques, referred to as computation or digital adaptive optics (CAO or DAO), can compensate the optical aberrations and can provide isotropic lateral resolution throughout the imaging volume without requiring any additional expensive adaptive optics hardware such as deformable mirrors, wavefront sensors etc. [7]. For the avoidance of confusion and for the sake of simplicity any numerical or computational post-processing methods for aberration correction from hereon will be referred to as DAO in this paper. Initial demonstrations were limited to *ex vivo* samples due to the motion related phase stability requirements of DAO [8–11]. However, soon thereafter several research groups demonstrated retinal photoreceptor OCT imaging under phase stability conditions aided by different DAO techniques [12–14]. Due to high imaging speed requirement of equivalent to > 10 volumes/second in order to avoid any detrimental effect of motion on phase, its demonstration was limited to only *en face* flying spot time domain (TD) OCT, line field spectral domain (LF SD) OCT or full field (FF) OCT. Kumar et al. demonstrated DAO based wavefront detection and correction for photoreceptor imaging using a 1060 nm swept source (SS) OCT system but with a very narrow field of view (FOV) limited by the A-scan rate provided by the swept source laser (SSL) [15].

All known DAO techniques with the exception of subaperture based DAO, which is the digital equivalent of Hartmann sensor, follow iterative approaches that try to optimize a defined image intensity-based metric. These techniques are computationally quite expensive and often not robust in case the image suffers from a complex wavefront error that requires fitting of Zernike modes of higher than 4^th^ order. They have the tendency to get stuck in local minima of the defined metric function as the chosen metric function may not be suitable according to the nature of the object scene and may fail to deliver the optimal result. Researchers have recently been trying to come up with techniques that can improve both speed and robustness of the optimization based DAO [16–18].

In this paper, a novel non-iterative DAO technique is presented in which the wavefront error is calculated using the phase difference between the pupil field and its digital copies translated by a pixel along the horizontal and vertical direction of the defined co-ordinate system of the pupil plane. Kumar has filed the patent describing the technique in 2018 [19]. This technique, which in principle is the digital equivalent of lateral shearing of wavefronts, is referred to as digital lateral shearing-based DAO (DLS-DAO). Unlike optimization-based DAO, this robust technique works directly with the slope of the wavefront error, independent of the intensity distribution in the image plane, and provides high computational speed without compromising the accuracy. The technique is useful in applications such as interferometric or digital holography based microscopy, metrology, and as digital wavefront sensor in adaptive optics, where focusing of light in the sample is involved that creates a guide star (GS) or where the sample itself exhibits GS-like structures. The theory of DLS-DAO is described in detail in section 2. Computer simulation and experimental results are provided in section 3 and 4 respectively and the results are also compared with the non-iterative subaperture-DAO technique. A DAO implementation for human photoreceptor imaging *in vivo* with a point scanning 1060 nm SS-OCT system at the eccentricity of 2 degrees from the fovea with a FOV that can allow reasonable image analysis has been demonstrated for the first time to our knowledge.

## 2. Theory

In interferometric imaging, the detected signal is given by

(1) $$I_{d}(\xi ,\eta ,t)=|E_{o}(\xi ,\eta ,t){|}^{2}+|E_{R}(\xi ,\eta ,t){|}^{2}+E_{o}(\xi ,\eta ,t)E_{R}^{*}(\xi ,\eta ,t)+E_{o}^{*}(\xi ,\eta ,t)E_{R}(\xi ,\eta ,t)$$

where $E_{o}$ and $E_{R}\;$are the images of the object/sample and the reference field at the detector plane respectively, and (ξ,η) are the coordinates of the detection plane in the spatial domain, which is conjugate to the sample or the object plane. The complex valued signal $E_{s}=E_{o}E_{R\;}^{*}$, that contains the phase information about the object in its conjugate detection plane, can be retrieved by using phase shifting techniques that modulate the interference signal in time *t*. This can be done for example by translating the reference mirror with a piezo-electric transducer, or by introducing an optical frequency shift in the reference light by using a moving grating or by using an acousto-optic modulator [1]. For standard TD OCT the simple reference arm scan introduces a modulation along the delay axis. The phase is then obtained by a simple Hilbert transform method [20]. In case of full field TD OCT or *en face* TD OCT the spatial carrier frequency can be introduced by tilting the reference mirror in an off-axis configuration, which separates the complex valued signal of interest $E_{s}=E_{o}E_{R}^{*}\;$from the complex conjugate term $E_{s}^{*}=E_{o}^{*}E_{R}$ in the spatial Fourier domain (FD), which can be then filtered out digitally [21]. In FD OCT system, the spectrum of the interference signal is recorded either spatially using a broadband light source and a spectrometer, or temporally by frequency sweeping the laser source. 1-D fast Fourier transform (FFT) of the signal along the frequency dimension, gives the complex valued signal for each depth in the sample [1]. Since the focus of the presented research work is OCT, we assume from henceforth that we have access to the complex valued data that contains phase information corresponding to each depth of the sample. The 2-D Fourier transform of the complex data corresponding to the *en face* plane at a given depth can be written as

(2) $$FT_{2D}[E_{s}(\xi ,\eta )]={\tilde{E}}_{s}(x,y)={\tilde{E}}_{ideal}(x,y)\text{exp}[i\phi_{e}(x,y)]$$

where ${\tilde{E}}_{s}$ is the Fourier transform of $E_{s\;}$, ${\tilde{E}}_{ideal}\;$is the ideal band-limited Fourier transform of the signal without any optical aberration, $\phi_{e}$ is the phase error, and (x,y) are the coordinates in the Fourier plane. We calculate the shifted version of$\;{\tilde{E}}_{s}$ shifted by small distance Δx and Δy along *x* and *y* respectively as

(3) $${\tilde{E}}_{s}(x+\Delta x,y)={\tilde{E}}_{ideal}(x+\Delta x,y)\text{exp}[i\phi_{e}(x+\Delta x,y)],\;\text{and}$$

(4) $${\tilde{E}}_{s}(x,y+\Delta y)={\tilde{E}}_{ideal}(x,y+\Delta y)\text{exp}[i\phi_{e}(x,y+\Delta y)].$$

If we consider the object to be point like, then we can write

(5) E(ξ,η)=Δ(ξ,η)A(ξ,η)exp(iφ(ξ,η))

where Δ(ξ,η) is the Kronecker delta function, *A* is the amplitude and φis the phase. In this case the Fourier transform of the ideal signal is given by

(6) $${\tilde{E}}_{ideal}(x,y)={\tilde{E}}_{ideal}(x+\Delta x,y)={\tilde{E}}_{ideal}(x,y+\Delta y)=A(0,0)\text{exp}[i\varphi (0,0)].$$

Multiplying ${\tilde{E}}_{s}(x+\Delta x,y)$ with the conjugate of ${\tilde{E}}_{s}(x,y)$ on a point-by-point basis and using Eqs. (5)–(6) we get

(7) $${\tilde{E}}_{s}(x+\Delta x,y){\tilde{E}}_{s}^{*}(x,y)=|A(0,0){|}^{2}\text{exp}\{i[\phi_{e}(x+\Delta x,y)-\phi_{e}(x,y)]\}$$

Taking the argument of Eq. (7), and using the first order Taylor expansion with respect to *x* yields

(8) $$∡[{\tilde{E}}_{s}(x+\Delta x,y){\tilde{E}}_{s}^{*}(x,y)]=\left[\phi_{e}(x,y)+\Delta x\frac{\partial \phi_{e}(x,y)}{\partial x}\right]-\phi_{e}(x,y).$$

Hence, we can find the slope $s_{x}$ of the wavefront error $\phi_{e}$ along *x* as

(9) $$s_{x}(\bar{x},\bar{y})=\frac{\partial \phi_{e}(\bar{x},\bar{y})}{\partial \bar{x}}=\left(\frac{1}{\Delta \bar{x}}\right)∡[{\tilde{E}}_{s}(\bar{x}+\Delta \bar{x},\bar{y}){\tilde{E}}_{s}^{*}(\bar{x},\bar{y})].$$

Similar, the slope $s_{y}$ of the wavefront error along *y* is given by

(10) $$s_{y}(\bar{x},\bar{y})=\frac{\partial \phi_{e}(\bar{x},\bar{y})}{\partial \bar{y}}=\left(\frac{1}{\Delta \bar{y}}\right)∡[{\tilde{E}}_{s}(\bar{x},\bar{y}+\Delta \bar{y}){\tilde{E}}_{s}^{*}(\bar{x},\bar{y})]$$

where $\left(\bar{x},\bar{y}\right)$ is the normalized the co-ordinate in the Fourier space such that the aperture lies within a circle of unit radius to make it suitable for Zernike polynomial fitting. We refer to the method of determination of slopes using Eqs. (9) and (10) as DLS-DAO. If the point spread function (PSF), corresponding to the focused Gaussian beam in the sample, is properly sampled according to the Nyquist criterion, then the resulting pupil after 2D FFT is band-limited with the Gaussian profile. The distance from the central peak intensity value position to the boundary where the intensity value drops to 1/e^2^ of the peak value is equal to the radius. Since the data is in digital format, the diameter of the pupil, which is twice the radius, can be measured in terms of number of pixels *M.* The normalized shearing in *x* and *y* is given by $\Delta \bar{x}=2M_{sx}/M$ and $\Delta \bar{y}=2M_{sy}/M$ respectively with diameter of the pupil as *M* pixels and shift in x and y as $M_{sx}\;$and $M_{sy}$ pixels respectively. Thus, using lateral shifts only in terms of pixels allows the slope information to be obtained without *a priori* knowledge of any other system parameters. In our computer simulations and experiments, we show that unit pixel shift for lateral shearing yields results very close to diffraction-limited performance with reasonable accuracy. Note that the unit pixel lateral shift also maximizes the number of slope data points for both $s_{x}\;$and $s_{y}$, which is of the order of $M^{2}$ for a pupil diameter of *M* pixels. The expressions for maximum and minimum detectable slope for unit pixel lateral shift are given in the Appendix A.

We can represent the phase error $\phi_{e}\;$in terms of orthogonal Zernike polynomials as

(11) $$\phi_{e}(\bar{x},\bar{y})=\sum_{i=1}^{P}a_{i}Z_{i}(\bar{x},\bar{y})$$

where $a_{i}$ is the coefficient of the *i*^th^ Zernike polynomial term $Z_{i}$. We compare the gradient of the phase error function with the calculated slope data and write the problem in matrix form as

(12) S=GA

where *S *= [*Sx*; *Sy*] with $S_{x}={\left[{\bar{s}}_{x,1},\ldots ,{\bar{s}}_{x,M^{2}}\right]}^{T}$ and $S_{y}={\left[{\bar{s}}_{y,1},\ldots ,{\bar{s}}_{y,M^{2}}\right]}^{T}$ as the column vectors containing normalized *x* and *y* slope components, $G=[\partial Z/\partial \bar{x};\;\partial Z/\partial \bar{y}\;]$ is the gradient matrix with $\partial Z/\partial \bar{x}$ and $\partial Z/\partial \bar{y}$ as $\left(M^{2}\times P\right)$ matrices of partial derivate of Zernike polynomials $Z_{i}(\bar{x},\bar{y})\;$with respect to $\bar{x}$ and $\bar{y}$, and $A={\left[a_{1},\ldots ,a_{P}\right]}^{T}$ is the vector containing the Zernike coefficients. We can estimate the least square solution for Eq. (12) as

(13) $$\hat{A}=(G^{T}G^{-1})G^{T}S.$$

The phase error can be calculated once the estimate of the coefficients can be determined using Eq. (13). The method described here for wavefront reconstruction using a set of basis functions and matrix formulation based on least square fit is referred to as Modal reconstruction. The basis function such as Taylor monomials or Fourier series can also be chosen instead of Zernike polynomials. The other method referred to as Zonal reconstruction involving least square fitting of the wavefront from the neighboring local slopes [22] can also be used.

**Fig. 1.**
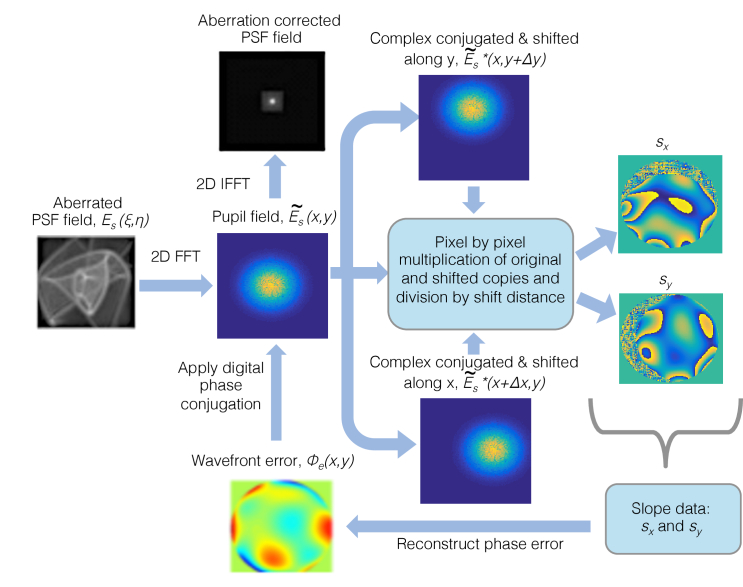
Schematic of the DLS-DAO algorithm.

Once the phase error is determined, the phase corrected image is obtained by first multiplying ${\tilde{E}}_{s}$ with correction factor$\;\text{exp}(-i\phi_{e})$ and calculating the inverse 2-D Fourier transform. The schematic of the DLS-DAO algorithm is shown in Fig. 1.

In our theoretical development, we have assumed our object to be point like and in the absence of any optical aberration it should ideally have a flat wavefront at the Fourier plane. Any deviation from this ideally flat wavefront can be detected with DLS-DAO. However, in the presence of an extended object, the ideal wavefront at the Fourier plane will not be flat and will vary with the spatial frequency depending on the nature of the object scene. The wavefront error detection using DLS-DAO will fail in that case. However, this method is still useful where focusing of light in the sample is involved, for example in point scanning microscopy, metrology and adaptive optics systems where focusing of light in the sample creates a pseudo guide star (GS), whose full 2D or 3D profile is detected based on either interferometry or digital holography. Also, for scene-based imaging, this method can be useful if a point scatterer in the sample acting as GS is present within the field of view (FOV). In case of retinal OCT, photoreceptors can be practically used as the natural GS for the DLS-DAO algorithm in order to detect the phase error. Once the phase error is determined, it can then be digitally phase conjugated with the calculated Fourier field of the aberrated full FOV *en face* photoreceptor layer of the retinal OCT. The correction assumes the FOV to lie within the same isoplanatic patch as the selected GS. Inverse 2D FFT of the corrected Fourier field can then provide an aberration free photoreceptor image.

## 3. Computer simulation

To simulate the aberrated image field of a point object or the point spread function (PSF), first the 2-D FFT of a matrix of size 512×512 pixels, representing an image, with center pixel value of unity and zero elsewhere is calculated. The calculated Fourier data is band-limited by multiplying it with a circular pupil of radius 128 pixels with pixel value of unity inside the radius and zero elsewhere. To introduce the wavefront error, the resultant data is multiplied with a phase error factor$\;\text{exp}[i\phi_{e}(\bar{x},\bar{y})]$ where $\phi_{e}(\bar{x},\bar{y})$ is calculated using Eq. (11). The coefficients of the Zernike polynomials of up to *P^th^* order are selected from normally distributed random numbers with mean value of 0.8 radians and standard deviation of 2 radians, and the overall phase is then scaled to get a predefined peak to valley (P-V) error. Finally, the 2-D IFFT of the result is calculated to get the simulated aberrated PSF.

**Fig. 2.**
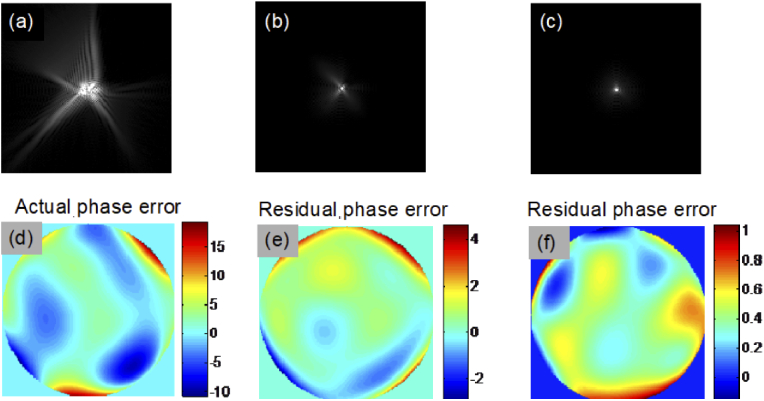
(a) Aberrated PSF affected by 6^th^ order wavefront error with P-V of 30 radians. (b) Phase corrected PSF obtained by subaperture-DAO. (c) Phase corrected PSFs obtained by DLS-DAO. (d) Actual phase error applied to get the aberrated PSF in (a). (e) and (f) are the residual phase error left after applying the sub-aperture DAO and DLS-DAO correction respectively.

**Fig. 3.**
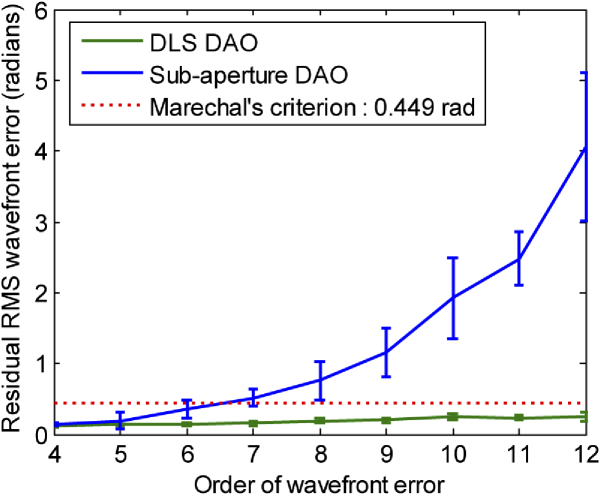
Plot of residual RMS wavefront error in radians left after correction by DLS- DAO and subaperture-DAO for increasing order of wavefront error with P-V of 20 radians.

Figure 2(a) shows the simulated PSF affected by 6^th^ order phase error with a P-V error of 30 radians as shown in Fig. 2(d). Figure 2(c) show the result of applying phase correction based on DLS-DAO with corresponding residual phase error map as shown in Fig. 2(f). The calculated residual root mean square (RMS) error value of 0.161 radians is well within the Marechal’s criterion of 0.449 radians for the diffraction-limited performance. The corresponding Strehl ratio value is 0.97, which is above the value of 0.8 required for diffraction limited performance in accordance with the Marechal’s criterion. We can clearly see that smearing has been reduced and the PSF is more tightly focused as compared to the PSF after subaperture-DAO correction as shown in Fig. 2(b), where some smearing of the PSF is still visible. Also, the residual phase error in case of subaperture-DAO is large as shown in Fig. 2(e) with residual RMS error value of 0.8311 radians. This is higher than the Marechal’s criterion of 0.449 radians for the diffraction limited performance. Also, the corresponding Strehl ratio value is 0.5, which is below the value of 0.8 required for the diffraction limited performance. Thus, from the results of the computer simulations shown in Fig. 2, we can deduce that DLS-DAO has a better performance than sub-aperture-DAO. The plots in Fig. 3 illustrate the superior performance of DLS-DAO, especially for higher order wavefront error. In our simulations for subaperture-DAO correction, 5 × 5 subapertures were used for 4^th^ -5^th^ order phase error, 7 × 7 subapertures were used for 6^th^ -8^th^ order phase error, 9 × 9 subapertures were used for 9^th^ -11^th^ order phase error, and 11 × 11 subapertures were used for 12^th^ order phase error estimation [9]. The residual RMS wavefront error is calculated after correction of the wavefront error by both DLS-DAO method and subaperture-DAO method. The wavefront error containing polynomial terms up to a given highest order was generated 10 times, and each time the P-V error was set to 20 radians and the coefficients of the wavefront error (see Eq. (11)), selected from normally distributed random numbers, were varied. The plots show the mean of residual RMS error, after correction by both methods, for the 10 trials and the error bars show the standard deviation. In general, the residual RMS error increases with increasing order of wavefront error. In case of subaperture-DAO the residual RMS error value increases from about 0.1443 radians for 4^th^ order to about 4.0639 radians for the 12^th^ order wavefront error, which is by far larger than Marechal’s criterion for the diffraction limited performance. However, the residual RMS error for DLS-DAO is well within the Marechal’s criterion of 0.449 radians for wavefront error order of up to 12. Hence, we can conclude that in our simulation the DLS-DAO method has yielded results comparable to diffraction-limited performance. The higher accuracy of wavefront error estimation using the DLS-DAO method is due to the fact that the number of slope samples depends on the total number of pixels within the aperture. For example, for pupil of diameter 256 pixels, we get >51000 samples of slope data for both *x* and *y* component in case of DLS-DAO with unit pixel lateral shift for any order of wavefront error. Whereas, in case of the subaperture-DAO method the number of samples of slope data is equal to the number of subapertures used. For example, for 7 × 7 sub-apertures, we get only 7^2^ = 49 samples of slope data for both *x* and *y* component.

Another limitation of subaperture-DAO, that becomes critical in real biological samples, appears when back reflected light might not be perfectly diffusive to fill the pupil uniformly, in which case the images from subaperture may not correlate well and can lead to error in slope estimation and subsequently in the wavefront error calculations. This causes effective loss in number of slope data [9]. From the theoretical formulation, it is clear that DLS-DAO does not suffer from such image correlation problem. Even though the number of slope data may be reduced for DLS-DAO in case the pupil is not filled completely, it would still be much higher than for the subaperture-DAO as each individual pixel in the pupil is used for calculation.

**Fig. 4.**
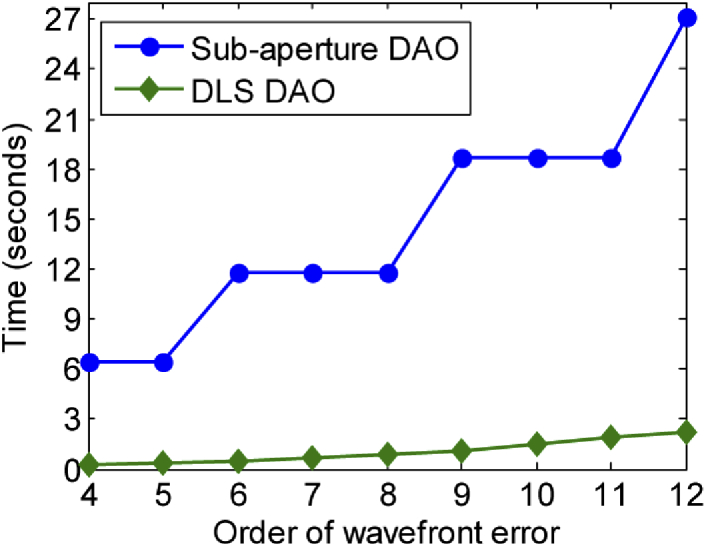
Comparison of computation time for DLS-DAO and subaperture-DAO for increasing order of wavefront error for 512 × 512 pixel image array (using CPU @ 3.30 GHz, 10 GB RAM).

In terms of computational speed, DLS-DAO is faster than the subaperture-DAO as it does not involve cross correlation of images from the subapertures. The computational complexity for subaperture-DAO increases with high order aberrations as the number of subapertures required for phase error estimation also increases. This is illustrated in Fig. 4: for a 512 × 512 pixel image array and 6^th^ order phase error correction, subaperture-DAO using 7 × 7 subapertures takes 11.8247 seconds on MATLAB (using CPU @ 3.30 GHz, 10 GB RAM), whereas DLS- DAO just takes 0.488 seconds. This indicates a huge improvement in computational speed by a factor of ∼24x for the DLS-DAO over the subaperture-DAO for 6^th^ order phase error correction. Furthermore for 12^th^ order phase error, subaperture-DAO using 11 × 11 subapertures takes 27.116 seconds, whereas DLS-DAO takes 2.252 seconds. Thus, for 12^th^ order phase error correction, there is still a significant improvement in computational speed for DLS-DAO over subaperture-DAO by a factor of ∼12x.

## 4. Experimental results

### 4.1 Ex vivo micro-beads phantom sample

The experimental set-up is similar to the one described in detail in ref. [15], which consists of a fiber based point scanning OCT system using a swept source laser (SSL) (AXSUN Tech., $\lambda_{\text{o}}=1060\;\text{nm},\;=110\;\text{nm}$) with sweep rate of 100 kHz. The measured axial resolution is 5 µm in air. The interference signal is detected by a dual balanced detector (DBD) (Thorlabs Inc., PDB430C) and digitalized at a rate of 250 M samples/s using a 12-bit analogue to digital converter (Alazartech Inc., ATS9360). A B-scan (OCT frame) rate of 250 Hz is achieved for a frame of size 400 (*x*) × 2560 (*z*) pixels. An OCT volume of size 400 (*x*) × 400 (*y*) × 2560 (*z*) is acquired using a X-Y galvo-scanner (Cambridge Technology) in the sample arm. The measured sensitivity of the system was 95 dB with 1.7 mW power on the sample. The beam was focused on the sample using a microscope objective (MO) lens (Thorlabs, LSM02-BB) with the effective numerical aperture (NA) of 0.13, resulting in a lateral resolution of 5 µm. A tilted glass plate was placed at the focal plane of a telescope in the sample arm to introduce the optical aberration.

**Fig. 5.**
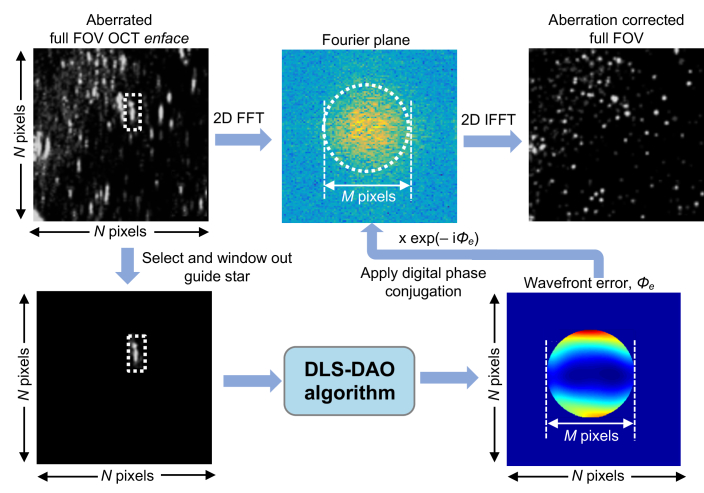
Schematic of the processing steps required to obtain aberration free full FOV image using GS selection and DLS-DAO algorithm. Schematic of DLS-DAO algorithm is shown in Fig. 1.

Figure 5 shows the schematic of the processing steps that was used to obtain the aberration correction over the whole FOV *en face* OCT image based on GS selection and DLS-DAO algorithm. First a GS, which represents the PSF of the system, is selected and windowed out from the full FOV of the aberrated enface OCT image of size *N *× *N* pixels, and zero-padded to the same original size of *N *× *N* pixels. This results in the pupil size in the Fourier plane due to the windowed GS being the same as the pupil size at the Fourier plane of the full FOV image. Note that the image should be properly sampled according to the Nyquist criterion so that the signal is band-limited, and the pupil is well defined in the Fourier plane. Also, the size of the window used for GS selection should be large enough to adequately capture the overall blur and smear, which depends on the strength, i.e. P-V or RMS value, and the order of the wavefront error. The windowed-out GS image is processed using the DLS-DAO that yields the wavefront error map, which is of same size with diameter of *M* pixels as the pupil obtained after 2D FFT calculation of the aberrated full FOV image. The calculated pupil field of the full FOV image is digitally phase conjugated with the wavefront error map obtained via DLS-DAO processing of the windowed-out GS. Finally, the 2D IFFT calculation of the resulting pupil field yields the aberration corrected full FOV image. Note that this method is based on the assumption that full FOV lies within the isoplanatic patch, where the aberration is the same throughout the FOV.

**Fig. 6.**
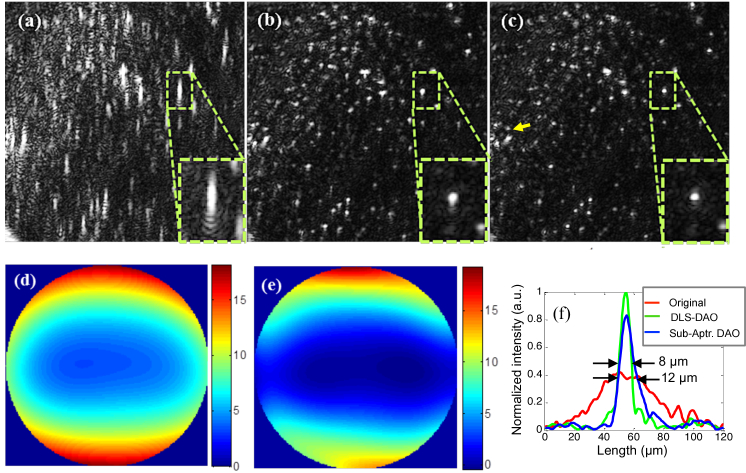
(a) Original OCT *en face* image of the microbeads phantom showing FOV of 875×875μm. (b) Image obtained by subaperture-DAO. (c) Image obtained by DLS-DAO. (d) Phase error estimation in radians by subaperture-DAO. (e) Phase error estimation in radians by DLS-DAO. (f) Profile plots across the GS before and after corrections.

Figure 6(a) shows the aberrated *en face* OCT image of the mono-layered micro-beads (mean diameter ∼10 µm) phantom sample acquired with the system. The micro-beads are smeared along the vertical direction due to the strong astigmatism introduced by the tilted glass plate. The GS was selected at the location of the green dotted box in Fig. 6(a). The image obtained by DLS-DAO (Fig. 6(c)) is more tightly focused as compared to the image obtained by subaperture-DAO (Fig. 6(b)) as the little bit of smearing left after sub-aperture-DAO correction is further reduced after DLS-DAO correction. For both methods the same GS is used and Zernike polynomials of up to 6^th^ order were fitted to the phase error. The zoomed-in view of the GS provided in the insets in Figs. 6(a)-(c) show the performance of the two methods. The profile plots across the GS (normalized by the peak value of the GS after the DLS-DAO correction) show that the profile has the smallest width after DLS-DAO correction. The full width at half maximum (FWHM) of the profile after subaperture-DAO correction is 12 µm, whereas after DLS-DAO correction it reduces to 8 µm. The value of the SNR for the original image is 33 dB, whereas after the subaperture-DAO and the DLS-DAO correction it increases to 38 and 41 dB respectively. Figure 6(d) shows the phase error estimate in radians obtained using subaperture-DAO method which contains dominant defocus and vertical astigmatism aberrations with corresponding Zernike coefficients of 0.28 and 0.33 waves respectively. Whereas the phase error estimation done by DLS-DAO, as shown in Fig. 6(e), has higher Zernike coefficient values of 0.45 and 0.47 waves for defocus and vertical astigmatism aberration terms respectively. The peak-to-valley error is 20 radians and the total RMS error is 0.71 waves. The claim that more accurate phase error estimation is done by DLS-DAO in comparison to subaperture-DAO is supported by better image quality both in terms of resolution and SNR enhancement. The total computational time taken by subaperture-DAO using MATLAB (on CPU @ 3.30 GHz, 10 GB RAM) was 3.2 seconds, whereas DLS-DAO method took 0.23 seconds, which is about 14 times faster.

Although, DLS-DAO correction has overall reduced the smearing of micro-beads quite uniformly, there are some regions at the edges, as the one marked by a yellow arrow in Fig. 6(c), where a slight but not so significant smearing is still visible. This may be due to the fact that the region is outside the isoplanatic patch and suffers from aberration that slightly differs from the one obtained using the GS, which may be due to change in off-axis aberration or due to change in turbulence/vibrations with time during the measurement. Region of interest (ROI) based DLS-DAO correction may be applied if the effect of anisotropic aberration is significant, which may provide more uniform correction over the full FOV [10].

### 4.2 Human retinal photoreceptor imaging in vivo

For human retinal photoreceptor imaging, a phase stable akinetic swept source laser (Insight Photonics Solutions Inc., $\lambda_{o}=1060\;$nm, Δλ=20 nm) with higher sweep rate of 450 kHz was used in order to achieve >1 kHz B-scan rate for a reasonable FOV so that any detrimental motion related phase instability could be avoided, which is critical for the performance of DAO correction [11]. Numerical phase correction was applied for residual motion correction as part of the OCT data post-processing [13]. A 200 × 200 pixel FOV was scanned at a B-scan rate of 1.1 kHz with 50% duty cycle. In order to achieve a higher NA of 0.2, the beam entering the subject’s dilated pupil was expanded to 7 mm. For pupil dilation one drop of mydriaticum “Agepha” 0,5% was applied to the eye prior to measurement. The power of the beam entering the eye was 1.8 mW, which is below the maximum permissible exposure (MPE) of 1.93 mW at 1060 nm wavelength [23]. Imaging was performed under the protocols approved by the ethics committee of the Medical University of Vienna. The measured axial resolution is ∼ 25 µm in air, which was sufficient to resolve between inner segment/outer segment (IS/OS) and retinal pigment epithelium (RPE) layers in retinal OCT tomograms. Note that the glass plate that was used to introduce astigmatism in the *ex vivo* experiment is now removed.

**Fig. 7.**
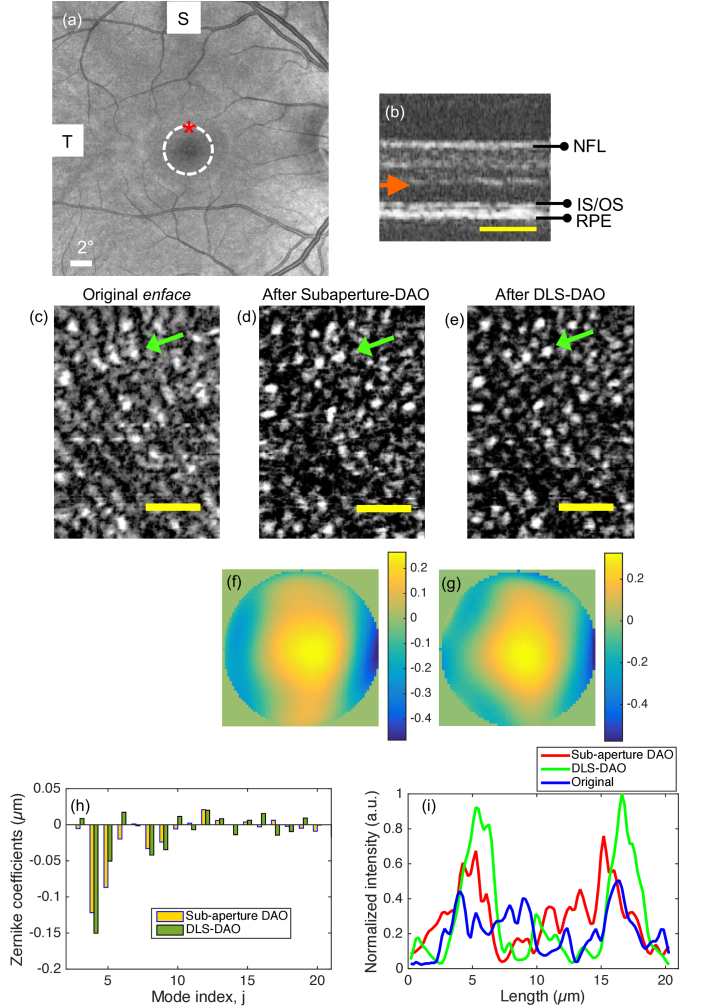
(a) SLO image of the retina with a FOV of 26°x26° in which white dotted circle is centred at the fovea with radius of 2° and red asterisk mark the location where OCT image is acquired. (b) B-scan (average of 10 frames) of the acquired retinal OCT with orange arrow showing the location of focal plane. (c) Original *en face* OCT image of the photoreceptor layer showing FOV of ∼0.15°x0.3° at an eccentricity of 2 degrees superior from fovea, (d) image after subaperture-DAO correction. (e) Image after DLS-DAO correction, (f) phase error map in microns calculated by subaperture-DAO. (g) Phase error map in microns calculated by DLS-DAO. (h) Plot of Zernike coefficients. (i) Profile plots across photoreceptors at the location marked by green arrows in (c)-(e). Yellow bar in (c)-(e) corresponds to 0.05 degrees. See **Visualization 1** to compare original photoreceptor image with images after subaperture-DAO and DLS-DAO correction.

Figure 7(a) shows a reference SLO image of the retina of a human subject with the FOV of 26°× 26° acquired with a commercial OCT system (Zeiss PLEX Elite 9000). Figures 7(b) and 7(c) show the OCT B-scan and the extracted *en face* image of the photoreceptor layer respectively, acquired at an eccentricity of 2 degrees superior from fovea, which is marked by red asterisk in the SLO image in Fig. 7(a). The *en face* image suffers from visible aberrations as photoreceptors appear smeared and difficult to resolve. Figure 7(d) shows the result after subaperture-DAO based correction where reduction in smearing and improvement in resolution is evident. However, the result after DLS-DAO correction, as shown in Fig. 7(e), shows superior performance as the smearing is even more reduced and individual photoreceptors appear more tightly focused throughout the FOV in comparison to the result after subaperture-DAO as shown in Fig. 7(d). The profile plots at the location where a GS was selected, marked by green arrows in Figs. 7(c)-(e), show the quantified improvements in the results as shown in Fig. 7(i). The profile peaks are narrower and higher after DLS-DAO correction, which implies the improvement in both resolution and SNR. The width of the peaks after DLS-DAO correction is ∼5 µm, which corresponds well with the mean photoreceptor size at 2 degrees from the fovea [23]. The calculated phase error maps using subaperture-DAO and DLS-DAO are shown in Fig. 7(f) and Fig. 7(g) respectively. In both cases Zernike terms up to sixth order were fitted in order to obtain the optimal result. The difference in the phase maps calculated by both methods is due to the difference in the calculated Zernike coefficient values for the different aberration modes. The plot of Zernike coefficients in Fig. 7(h) shows that defocus and vertical astigmatism are the dominant aberration modes. The calculated RMS wavefront error values by DLS-DAO and subaperture-DAO are 0.2 µm and 0.16 µm respectively. The total computational time to process a 200 × 200 pixel *en face* image frame with pupil of diameter 60 pixels by subaperture-DAO method (using MATLAB on CPU @ 3.30 GHz, 10 GB RAM) is 0.9 seconds, whereas by the DLS-DAO method it is only 0.071 seconds, which is an improvement by 12.7 times in speed.

**Fig. 8.**
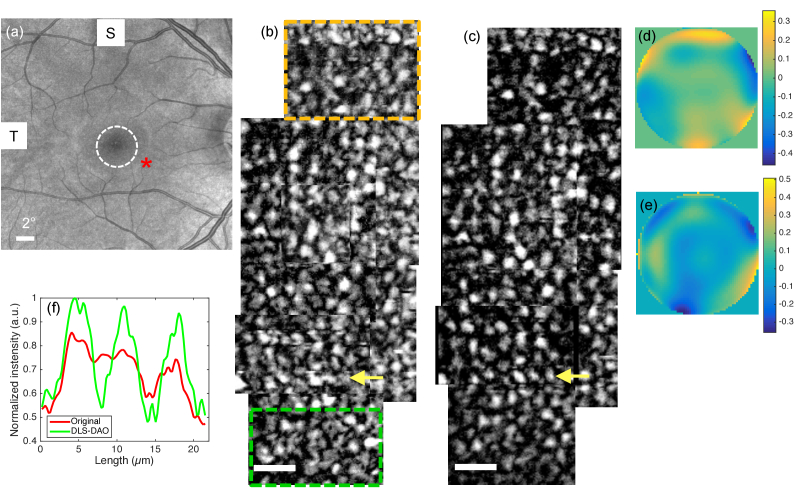
(a) SLO image of the retina with a FOV of 26°×26° in which white dotted circle is centered at fovea with radius of 2° and red asterisk marks the location at which OCT images are acquired. b) Mosaic of photoreceptor frames without any DAO correction. (c) Mosaic of the photoreceptor frames after DLS-DAO correction. (d) and (e) are the phase error maps with the color bar in microns corresponding to the region marked by the yellow and the green dotted box in (b). (f) Profile plots across the photoreceptors marked by the yellow arrows in (b) and (c). Scale bar in (b) and (c) represent 0.05 degrees.

In order to achieve a larger FOV, nine OCT volumes acquired at a location centered at 2.5 degrees inferior from the fovea were used. *En face* frames, that were extracted from OCT volumes by summing photoreceptor layers, were manually stitched in order to create a larger FOV. Only frames that were free from motion and overlapping with each other were used for stitching. Frames with overlapping regions showed change in defocus and other aberrations, which may be due to the subject’s changing visual accommodation between OCT volume acquisitions. Each region was separately processed using DLS-DAO algorithm by selecting a GS within that region and subsequently compensating for the phase error specific to that region in order to achieve the best performance. Figure 8(a) shows a reference SLO image of the retina with a FOV of 26°×26° in which a white dotted circle is centered at fovea indicates an eccentricity of 2° and the red asterisk marks the location at which OCT images are acquired. Figure 8(b) shows the original mosaic of stitched frames suffering from aberration, which degrades the image quality and makes it difficult to resolve individual photoreceptors clearly. After the DLS-DAO correction the mosaic appears in focus with individual photoreceptors clearly resolvable, as shown in Fig. 8(c). The total resulting FOV of the mosaic is 0.2 degrees (horizontal)×0.6 degrees (vertical), which is double in size in the vertical dimension as compared to the single scanned FOV. Figures 8(d) and 8(e) are the phase error maps that were calculated for the region marked by the yellow and the green dotted box respectively in Fig. 8(b). Zernike terms up to 8^th^ order (45 terms) were used in the phase error calculations, which provided better improvement in terms of photoreceptor resolution after correction as compared to 6^th^ order Zernike polynomial fitting as in the previous case shown in Fig. 7. The calculated RMS values for the phase error in Figs. 8(d) and 8(e) are 0.12 µm and 0.15 µm respectively. Figure 8(f) shows the normalized profile plot across the photoreceptors marked by the yellow arrow in Figs. 8(b) and 8(c). The profile plot after DLS-DAO correction shows clearly resolvable peaks corresponding to the photoreceptor pattern with mean peak to peak distance of ∼6 µm which corresponds well with the mean human cone spacing in the region between 2 and 3 degrees from fovea [24]. Also, the calculated mean cone packing density is ∼32,000 cones/mm^2^, which is also within the range reported in ref [24]. The small FOV of a single scan is due to the limitation imposed by the motion related phase stability criterion that requires high scan speed for DAO correction which in return is limited by the currently available sweep rate of SSL and galvo-scanner frequency. Nevertheless, the result in Fig. 8 demonstrates that it is feasible to overcome this limitation and achieve larger FOV by stitching photoreceptor frames obtained from overlapping OCT volumes.

## 5. Conclusions/discussion

The results in sections 3 and 4 confirm that DLS-DAO provides both high accuracy and computational speed. With unit pixel lateral shift between the original and the digitally shifted copies, the slope per pixel can be calculated. This yields a wavefront error map with high resolution and also allows higher order Zernike modes (> 8^th^ order) fitting for complex wavefront error calculation. Dynamic range, which relates to P-V and RMS values of the wavefront error, and sensitivity, which relates to the precision and accuracy, of the DLS-DAO method are ultimately limited by the system parameters such as pixel pitch in the pupil plane, number of pixels in the pupil and phase noise as defined by Eqs. (14) and (15) in appendix A. Nevertheless, using a point scanning SS-OCT system and a micro-beads phantom sample, a wavefront error with a high P-V value of 20 radians and RMS value of 0.71 waves (equivalent to 0.75 microns at 1060 nm wavelength) has been detected and corrected to achieve close to diffraction limited imaging performance. This demonstrates that the method can offer sufficiently high dynamic range for successful implementation in a general human population where the mean RMS value for total higher order aberration is around 0.3 microns [25]. Computer simulation and experimental results demonstrate that DLS-DAO provides better accuracy in comparison to subaperture-DAO, especially in case of higher order wavefront error. Also, the results after DLS-DAO corrections are closer to the diffraction limited performance, which indicates better precision or sensitivity. Non-iterative DAO methods have been shown to achieve similar or better performance in terms of dynamic range as published for iterative methods [16–18], i.e. with respect to P-V and RMS error values. However, in detail research needs to be done in the future in order to compare their accuracy or sensitivity with DLS-DAO with the same system parameter settings, especially in case where the wavefront error is represented by higher order Zernike terms. In the current study, a unit pixel shift was used in the DLS-DAO algorithm to achieve the maximum dynamic range for the given pixel pitch size in the pupil plane (see Appendix A, Eq. (14). However, the pixel pitch size in the pupil plane can be reduced to further increase the dynamic range by zero-padding the image array to a bigger array size, which leads to interpolated pupil data with increased number of pixels after 2D FFT. A unit pixel shift in this case will in effect be equivalent to sub-pixel shift with respect to the original data set. The resulting increase in achievable dynamic range would however come at the expense of reduced sensitivity, accuracy and the computational speed.

Simple calculation steps required in the DLS-DAO algorithm enable high speed of wavefront error calculation. In comparison to subaperture-DAO, which is also a non-iterative method, DLS-DAO offers more than 10x improvement in computational speed. Based on the recent publications, it also outperforms faster optimization-based DAO algorithms considering similar image size and computational platforms (i.e. MATLAB on CPU). For example DLS-DAO, after the GS is selected, takes only ∼0.5 seconds for a 512×512 pixels image with 6^th^ order Zernike mode fitting, whereas stochastic parallel gradient descent (SPGD) algorithm presented in ref. [18] takes > 2 seconds, while fitting only up to 4^th^ order Zernike modes (12 terms). Even though automatic GS selection and the need for automatic defocus correction [26] in case a GS is hard to resolve due to strong defocus blur can add to some computational complexity, the total processing time for a 512×512 pixels image with 6^th^ order Zernike mode fitting is still expected to be below 1 second and still be able to provide faster computational speed compared to the state-of-the-art iterative DAO methods. Nevertheless, in detail future study is needed to compare DLS-DAO with the state-of-the-art non-iterative methods for the same system parameter settings and sample.

DLS-DAO implemented on a SS-OCT system using a high-speed phase stable akinetic SSL enabled photoreceptor imaging at a B-scan rate of >1 kHz with a larger FOV, which supports better analysis of photoreceptor size and structure. While imaging, the focus was empirically set close to the photoreceptor layer by axially adjusting the ocular lens position in front of the eye until the IS/ OS and the RPE layer appeared brightest in the live B-scan view. The photoreceptors, although blurred due to residual defocus, were still quite distinguishable and could be selected as GS for the successful implementation of the DLS-DAO algorithm. For the current proof of principle demonstration of DLS-DAO, photoreceptors as GS were selected empirically. Future development and implementation will involve automatic GS selection based on suitable criteria.

Although, in most practical situations, people with expertise in OCT imaging can always roughly set the focus at a depth layer by visually evaluating the live B-scan view while changing the ocular lens position, situations might occur where the defocus is large enough to completely blur out the photoreceptors wherein selection of a GS is no longer feasible. In this case, defocus can be corrected using a fast two-subaperture refocusing method [26] as a first step that can allow photoreceptors to become distinguishable again. They can subsequently be used as GS for DLS-DAO processing to further reduce the residual defocus and other higher order aberrations [13].

One of the goals of the current study was also to image photoreceptors closer to fovea with a 1060 nm SS OCT system, which is quite challenging. This required increasing the NA to 0.2 (∼7mm beam at pupil), which resulted in a very small depth of field (DOF) of ∼13 µm in the tissue and led to significant loss of SNR beyond the DOF due to the confocal gating by the fiber-based detection. Although photoreceptors could be recovered and resolved using the DLS-DAO technique when the focus was set closer to the RPE layer, loss of SNR was too high to recover any significant structural information in *en face* layers beyond a few multiples of the DOF. A technique that utilizes dynamic focusing in depth could possibly help to obtain 3-D volume image with more detailed structural information of retina near fovea in the future. However, note that at lower NA, when the loss of SNR within a depth of ∼200-250 µm (typical thickness of retina) is not that significant and the optical aberrations other than defocus are the same throughout the volume, it may be possible to obtain aberration free volume image by applying the phase correction known from the DLS-DAO processing of photoreceptor layer together with numerical depth dependent defocus correction. Ginner et al. have demonstrated volumetric aberration correction in retinal OCT volumes using subaperture-DAO, at eccentricities of 4 degrees or more from the fovea [13]. The DLS-DAO method is expected to work similarly at such higher eccentricities from the fovea where the photoceptors are larger in size and resolvable with a lower NA.

Further increasing the FOV uniformly along both lateral dimensions by utilizing a SSL with even higher sweep rate and stitching of photoreceptor frames with proper target-visual alignment enabled with an eye tracking system could be another next step of the future development. Nevertheless, in spite of the current limitations, DLS-DAO-enabled human photoreceptor imaging *in vivo* with a point scanning SS-OCT system at 1060 nm has been demonstrated for the first time to our knowledge closer to fovea at eccentricities of 2 and 2.5 degrees with a FOV that can allow reasonable image analysis. Also, the presented DLS-DAO technique can potentially find application in digital holographic microscopy, *en face* FF-OCT and as wavefront sensor for HAO-OCT and clinical aberrometry.

## Appendix A

It is evident from Eqs. (9) and (12) that the maximum slope, which determines the dynamic range of the wavefront error detection, is detected at the minimum pixel shift of unity. Assuming the phase difference per pixel between the original pupil field and the lateral shifted digital copies is within the –π to +π range to avoid phase wrapping (phase modulo 2π), the maximum slope ${\text{s}}_{\text{max}}$ for phase error expressed in waves without any normalization factor can be deduced as

(14) $$s_{\text{max}}=\frac{\lambda }{2\Delta_{x}}=\frac{\lambda M}{2D}$$

where λ is the wavelength of light, Δx is the pixel pitch in the pupil plane and *D* is the diameter of the limiting pupil. Hence the dynamic range increases with the increase in the resolution of the pupil data. Whereas, for the unit pixel lateral shift, the minimum detected slope $s_{\text{min}}$, which determines the sensitivity of the wavefront error detection, is given by

(15) $$s_{\text{min}}=\frac{\lambda \sigma_{N}M}{2\pi D}$$

where $\sigma_{N}$ is the standard deviation of the phase noise in radians in the pupil plane.
